# Modified Bunnell suture repair versus bundle-to-bundle suture repair for acute Achilles tendon rupture: a prospective comparative study of patients aged <45 years

**DOI:** 10.1186/s12891-020-03588-5

**Published:** 2020-08-26

**Authors:** Xiaomeng Wang, Huixin Liu, Dengke Li, Zixuan Luo, Yansen Li, Fengqi Zhang

**Affiliations:** 1 Hebei Medical University Third Affiliated Hospital, 139 Ziqiang Road, Shijiazhuang, 050051 Hebei China; 2 Hebei Province General Hospital, 384 West Heping Road, Shijiazhuang, 050000 Hebei China

**Keywords:** Achilles tendon repair, Modified Bunnell suture, Bundle-to-bundle suture, Achilles tendon rupture

## Abstract

**Background:**

This study aimed to compare the operative outcome of percutaneous repair (modified Bunnell suture technique) versus open repair (bundle-to-bundle suture technique) of acute Achilles tendon rupture.

**Methods:**

Seventy-two consecutive patients who underwent surgical treatment of Achilles tendon rupture were evaluated in this prospective study. Thirty-six patients were treated using the bundle-to-bundle suture technique (group A), and 36 patients were treated using the modified Bunnell suture technique (group B). All patients underwent functional examination comprising measurement of the calf muscle circumference and performance of the single-leg heel-rise test. The length and diameter of the Achilles tendon were compared between the injured and uninjured sides on magnetic resonance imaging. The number of single-leg heel rises (height > 5 cm) performed within 15 s was compared between the injured and uninjured sides. The ankle range of motion was also recorded. The Achilles tendon total rupture score (ATRS), American Orthopaedic Foot and Ankle Society (AOFAS) ankle-hindfoot scale score, and visual analog scale (VAS) pain score were used to evaluate the clinical outcome at 12 months postoperatively.

**Results:**

A total of 61 patients were followed up. The mean follow-up duration did not significantly differ between group A (23.73 ± 2.81 months) and group B (22.61 ± 3.96 months). However, there were significant differences between groups in the heel-rise test (group A, 1.74 ± 0.96; group B, 2.37 ± 1.42) and length of the Achilles tendon (group A, 11.98 ± 1.64 cm; group B, 11.11 ± 1.74 cm). The calf circumference of the injured side was significantly larger in group A than in group B (*p* = 0.043). The cross-sectional diameter of the Achilles tendon was significantly smaller in group A than group B. At final follow-up, there were no significant differences between the two groups in the ATRS, AOFAS score, or VAS score. One patient in group A had delayed wound healing, which resolved in 40 days.

**Conclusions:**

Patients with acute Achilles tendon rupture treated with open repair (bundle-to-bundle suture technique) achieved a better clinical outcome regarding the heel-rise test and calf circumference compared with those treated with percutaneous repair (modified Bunnell suture technique).

**Trial registration:**

Chinese Clinical Trial Registry, ChiCTR2000035229, 8/4/2020, Retrospectively registered.

## Background

Although the Achilles tendon is the strongest tendon in the body, it is still prone to rupture [1]. The incidence of Achilles tendon rupture is 6 to 18 in every 100,000 individuals, and has increased in the last few decades [2]. The horsetail-like ends of the ruptured Achilles tendon are often located 2 to 6 cm above the tendon attachment point on the calcaneus [3].

Many Achilles tendon repair techniques have been described. Achilles tendon rupture is treated using non-operative treatment, open repair, percutaneous repair, or minimally invasive repair techniques [4–7]. However, non-operative treatment results in a high re-rupture rate [8], and healing in a lengthened position may result in loss of calf muscle strength [9]. Open repair is associated with surgical complications, such as skin–tendon adhesions, infection, delayed healing of the surgical wound, and suture granulomas [10]. Compared with open repair, percutaneous repair of Achilles tendon rupture reportedly reduces the destruction of the blood supply and lowers the risk of wound complications and infections [11]. However, gaps may be created in the area of percutaneous repair, which can lead to postoperative tendon weakness and granulation hyperplasia. Newer open repair techniques achieve reduced complication rates and more successful outcomes compared with traditional open surgery [7, 12, 13]. For example, the bundle-to-bundle suture technique is a type of open repair that minimizes the loss of Achilles tendon length and restores good ankle function [7, 12, 13]. The closure and restoration of the paratenon and fascia cruris aims to optimize blood flow to the repaired Achilles tendon [14].

The objective of the present study was to compare the outcome of open repair (bundle-to-bundle suture technique) with percutaneous repair (modified Bunnell suture technique) for acute Achilles tendon rupture. The hypothesis was that the bundle-to-bundle suture technique would achieve a better clinical outcome than the modified Bunnell suture technique.

## Methods

### Study population

This was a prospective, quasi-randomized, comparative trial that enrolled all patients who were surgically treated for acute Achilles tendon rupture from May 2016 to January 2018 (surgery was performed less than 7 days after injury). The trial was registered in a WHO registered trial registry(ChiCTR2000035229). Achilles tendon rupture was diagnosed by clinical examination (Thompson test) and magnetic resonance imaging (MRI) in each patient [15].

The inclusion criteria were (1) age 18 to 45 years, (2) body mass index (BMI) < 34 kg/m^2^, (3) closed injury, and (4) acute Achilles tendon rupture (< 7 days since tendon rupture). The exclusion criteria were (1) partial Achilles tendon rupture (diagnosed on MRI or via intraoperative probing), (2) Achilles tendon ruptured at a distance of less than 2 cm from the tendon insertion point, and (3) diseases that may affect the results of the functional tests, such as autoimmune disease, deep vein thrombosis, or neuropathy.

All patients provided written consent to participate in the study and return for standardized follow-up examinations at 1, 3, 6, and 12 months postoperatively. The operative technique was selected by assigning each patient an odd or even number in accordance with the order of hospital admission.

The same surgeon (F.Q.Z.) performed the preoperative physical examinations and operations for all patients. Open repair (bundle-to-bundle suture technique) was performed in group A (*n* = 30), while percutaneous repair (modified Bunnell suture technique) was conducted in group B (*n* = 31).

### Surgical procedures

#### Bundle-to-bundle suture technique

**Fig. 1:**
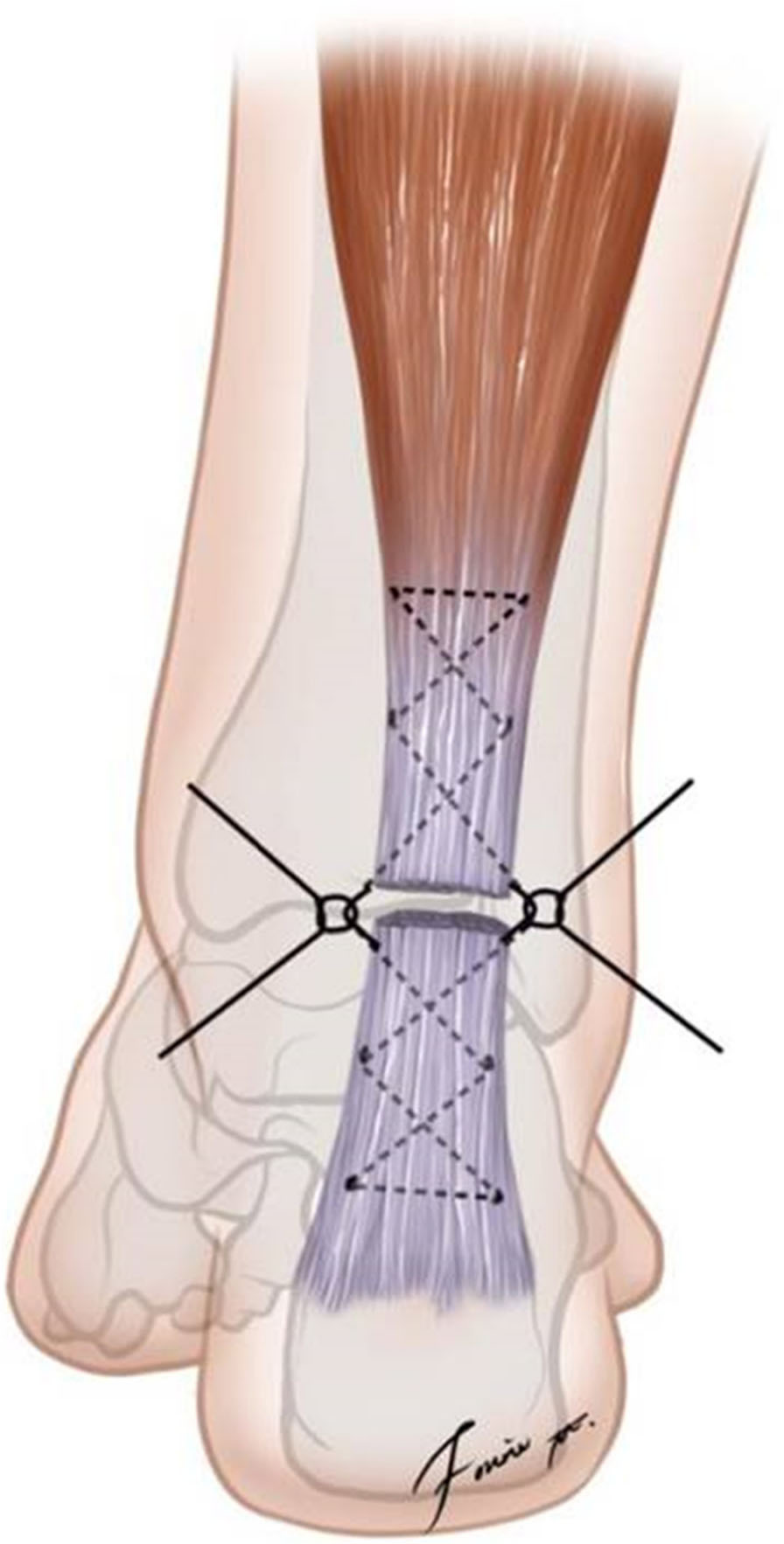
Diagrammatic drawing of the Achilles tendon sutured using open repair (bundle-to-bundle suture technique)

The posteromedial Achilles tendon approach was used, with the site of Achilles tendon rupture at the center of the incision. After the ruptured part of the Achilles tendon was exposed, the congested tissue was carefully removed, and the ruptured Achilles tendon was shifted to expose the deep soft tissue. The deep soft tissue was sutured first, and the soft tissue bed (deep fascia and muscle epimysium) of the Achilles tendon was then repaired to aid in the recovery and healing of the blood supply to the Achilles tendon. The tendon band of the superficial proximal gastrocnemius was turned laterally and sutured to the band of the distal lateral end of the Achilles tendon, while the tendon band of the deep proximal soleus was turned inward and sutured to the band of the distal medial end of the Achilles tendon. The horsetail-like ends of the ruptured Achilles tendon were aligned in accordance with the anatomical characteristics of the Achilles tendon, and an anatomical fascicle repair was performed. The Achilles tendon was typically divided into 20–30 bundles, and the proximal and distal portions of the adjacent avulsed, fascicular bundles of the Achilles tendon were repaired from the deep aspect to the lamina; absorbable 4–0 sutures were used to connect the ends, and 5–0 Prolene was used to reinforce the repair. The long tendon band was sutured to the short tendon band using 4–0 Prolene to avoid the creation of a short, retracted Achilles tendon and excessive plantarflexion of the ankle. Nonabsorbable 4–0 suture was used to suture the outer membrane of the Achilles tendon, deep fascia, subcutaneous tissue, and skin. This bundle-to-bundle suture technique was first introduced by Zhao et al. [14] (Fig. 1).

#### Modified Bunnell suture technique

**Fig. 2:**
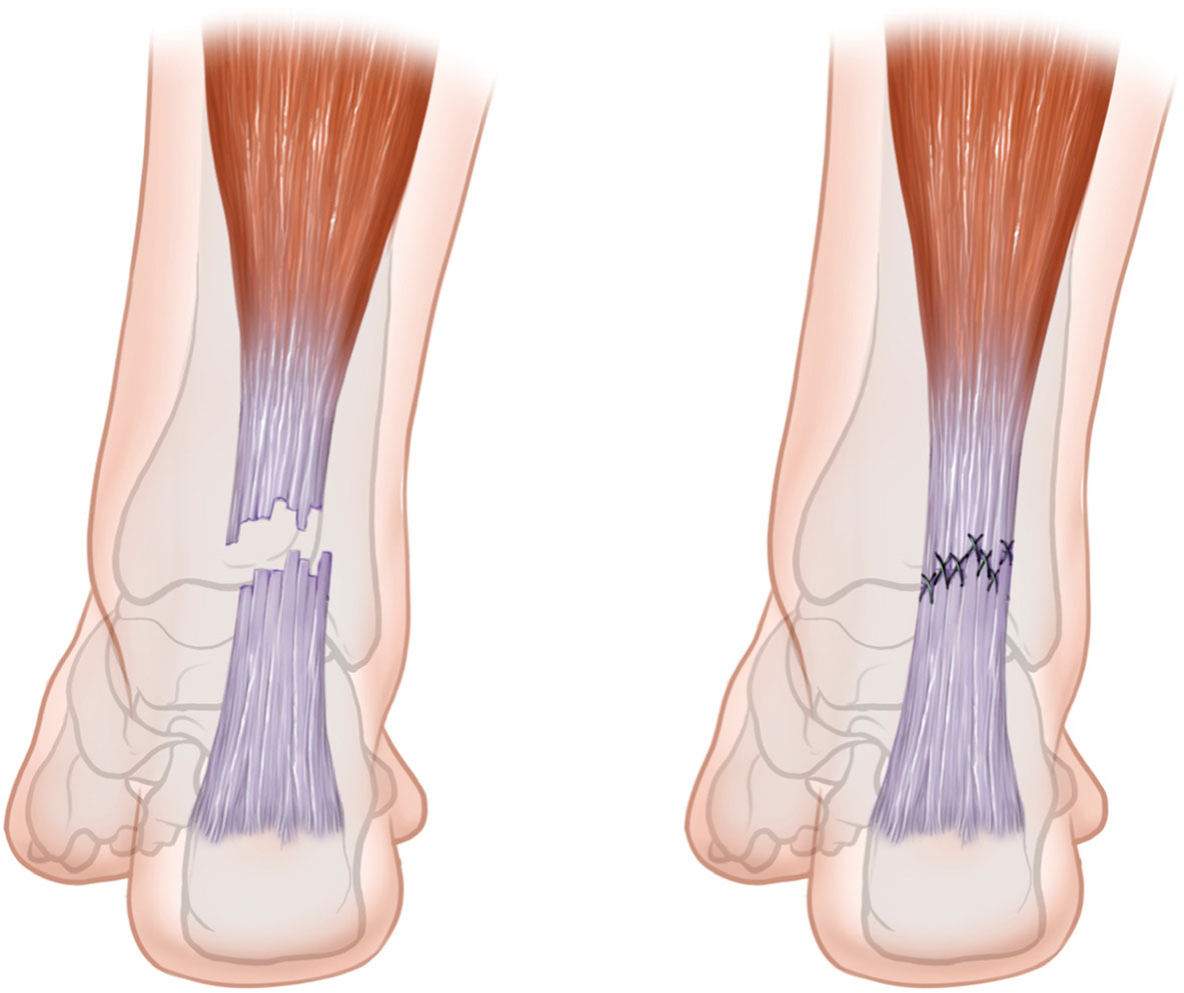
Diagrammatic drawing of the Achilles tendon sutured using percutaneous repair (modified Bunnell suture technique)

One mark was made on the skin in the middle of the depression of the ruptured Achilles tendon, and two or three marks were made on both sides of the distal and proximal Achilles tendon. Approximately 11 to 13 small 1-cm incisions were made at each mark to reach the deep fascia. PDS-II suture was threaded through the distal incision, passed through the tendinous tissue, and then threaded through the central incision. Next, a “Z” suture was performed by threading the PDS-II through the proximal incision and then through the central incision. The distal and proximal sutures were tightened to join the ruptured ends of the Achilles tendon, and the strength of the join was assessed using the Thompson test. The transverse incision was sutured, and the stab incisions were closed. The sural nerve was protected during the performance of all incisions. This modified Bunnell suture technique was first introduced by Ma and Griffith [16] (Fig. 2).

### Postoperative rehabilitation

The postoperative rehabilitation was performed by an experienced physical therapist. The affected limb was lifted up, and the quadriceps femoris and triceps crus were exercised by isometric contraction and relaxation on the second postoperative day. The skin sutures were removed at 2 weeks postoperatively. Partial protected weightbearing with walking boots was gradually allowed until the patients were able to walk with a partial load at 4 weeks postoperatively; the partial load was then changed to a full load within 4 to 6 weeks postoperatively. Four layers of wedge insoles were placed inside the walking boots, and one layer was removed every l to 2 weeks. Flexion and extension of the ankle was allowed in the neutral position from 6 weeks postoperatively. After 2 weeks of full weightbearing exercise with walking boots, the walking boots were removed. Walking with sneakers and functional ankle range-of-motion exercise were allowed from 2 months postoperatively, and heel-lifting functional exercise was allowed from 10 weeks postoperatively.

### Functional evaluation

The patients attended follow-up visits at 1, 3, 6, and 12 months postoperatively. The function was evaluated using both a functional examination and patient-reported outcome measures (PROMs). The functional examination findings and clinical scores were evaluated at 12 months postoperatively.

The functional examination consisted of the following items: measurement of the calf circumference, measurement of PROMs [17], measurement of the Achilles tendon width and anteroposterior dimension, and the heel-rise index of the single-leg heel-rise test. All postoperative evaluations were performed by nonsurgical personnel who were unaware of the patients’ surgical procedures.

Active plantarflexion and dorsiflexion of the ankle was measured with the ankle at 5 degrees of plantar flexion. These ranges were measured by a goniometer with the patient in supine position with the knee extended.

The diameter and length of the Achilles tendon on the injured side were measured on T1- and T2-weighted MRI with normal and fat suppression techniques. The patient sat with their leg horizontal and the foot placed in a frame. The length of the Achilles tendon was defined as the mid-sagittal length from the most distal end of the soleus insertion to the proximal portion of the calcaneal insertion. The diameter of the Achilles tendon was defined as the maximum diameter of the Achilles tendon in the axial plane.

The calf circumference at 25 cm above the tibial malleolus on the injured side was compared with the calf circumference at the same point on the uninjured side. The heel-rise index was defined as the number of single-leg heel rises to a height of > 5 cm performed in 15 s using the injured side compared with the uninjured side [18]. The height of the heel-rise test was monitored by one person.

The PROMs were the Achilles tendon total rupture score (ATRS) [19], American Orthopaedic Foot and Ankle Society (AOFAS) ankle-hindfoot scale score [20], and visual analog scale (VAS) pain score.

### Statistical analysis

Statistical analysis was performed using SPSS version 21.0 (IBM Corp., Armonk, NY, USA). The demographic data and all outcome parameters were tested for deviation from the normal distribution. Differences between the injured and uninjured sides were tested with the two-tailed, unpaired t-test. Differences between the two treatment groups in age, sex, BMI, and follow-up duration were examined using the paired-samples t-test and Fisher’s test for continuous variables, and using the χ^2^ test for categorical data. Differences between the two treatment groups in the ATRS, AOFAS score, and VAS score were determined with an unpaired-samples t-test or the Mann-Whitney rank sum test. Pearson correlation analysis was performed during the functional examination of the patients. A *P* value of < 0.05 was considered statistically significant.

## Results

**Fig. 3:**
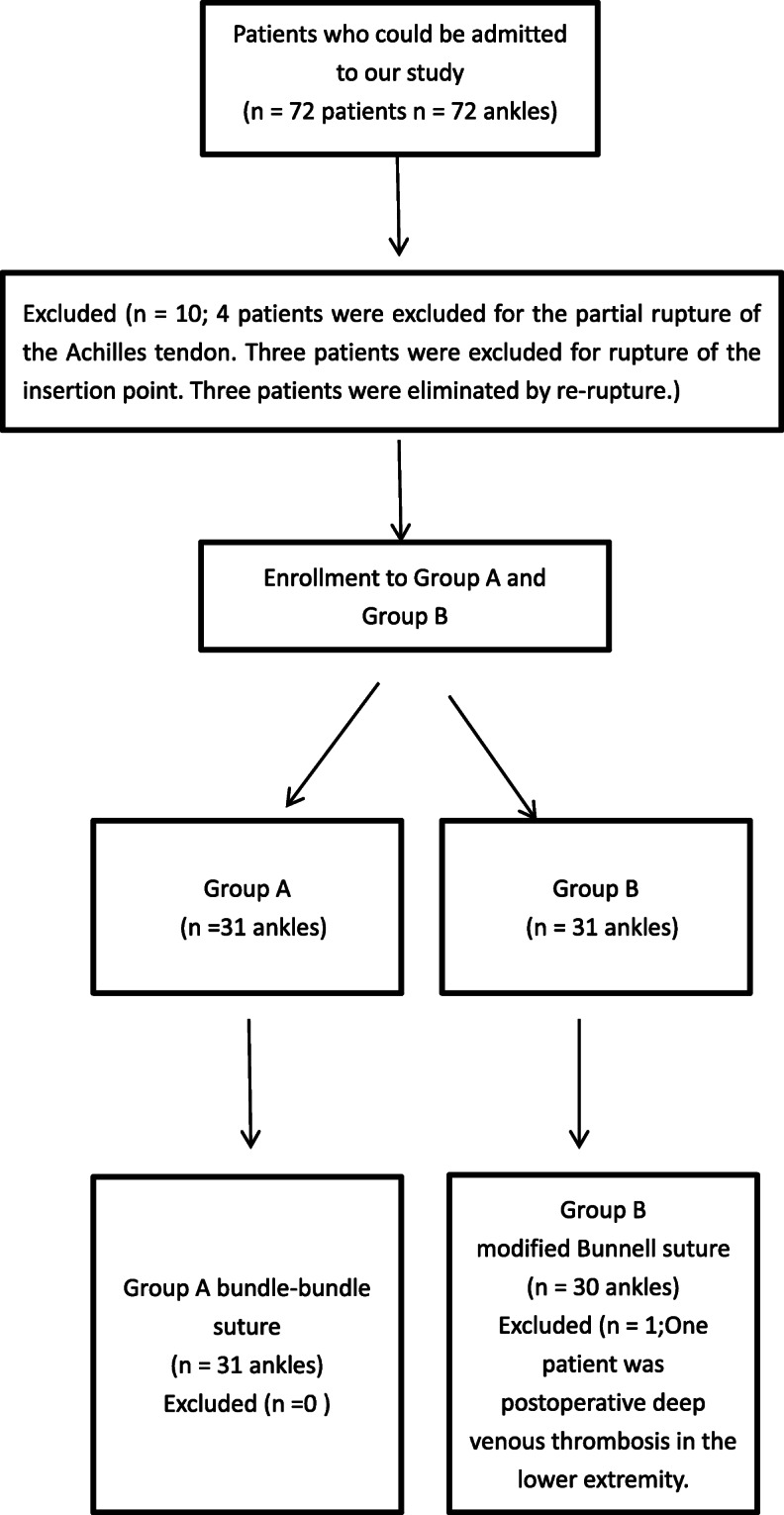
Patient selection flowchart

Seventy-two patients participated in the study, which continued for a total duration of 19 months. Eleven patients were excluded due to partial rupture of the Achilles tendon (*n* = 4), rupture of the Achilles tendon insertion point (*n* = 3), re-rupture of the Achilles tendon (*n* = 3), and postoperative deep venous thrombosis of the lower extremity (*n* = 1). A final total of 61 patients were included in this clinical study. The CONSORT flow chart of patient selection is shown in Fig. 3. There were no significant differences between the two groups in age, sex, or BMI. The patient demographic characteristics are shown in Table 1.

**Table 1:** Patient demographics

	Group A(bundle-bundle repair)	Group B(modified Bunnel suture)
Age	41.46 ± 1.59	40.06 ± 1.82
Sex(Male/Female)	28/2	28/3
BMI	24.21 ± 3.26	24.37 ± 3.23

^“*”^:statistically significant difference between two groups (*P* > 0.05; Student’s t-test)

There was no difference between groups A and B in the mean follow-up duration (23.73 ± 2.81 months vs. 22.61 ± 3.96 months, respectively). No patient in either group had a negative heel-rise index at final follow-up. Compared with group B, group A had a significantly smaller heel-rise index (1.74 ± 0.96 vs. 2.37 ± 1.42; *P* = 0.000) and a significantly longer Achilles tendon (11.98 ± 1.64 cm vs. 11.11 ± 1.74 cm; *P* = 0.048). Groups A and B did not significantly differ in the range of dorsiflexion (19.06 ± 2.42 degrees vs. 19.25 ± 3.24 degrees, respectively) or range of plantarflexion (36.58 ± 4.39 degrees vs. 35.41 ± 4.45 degrees, respectively) at the final evaluation. The calf circumference was smaller on the injured side than on the uninjured side in both groups. Group A had a significantly larger calf circumference on the injured side than group B (*P* = 0.043). There was no correlation between the calf circumference and the heel-rise index. The postoperative cross-sectional diameter of the Achilles tendon was significantly smaller in group A than group B (*P* = 0.000) (Table 2). The two groups did not significantly differ in the ATRS, AOFAS score, or VAS score (Table 3). No patient in either group developed postoperative complications such as infection, sural nerve lesion, or Achilles tendon re-rupture. However, one patient in group A experienced delayed wound healing, which resolved in 40 days. All bacterial cultures were negative.

**Table 2:** Functional outcome after percutaneous repair versus open repair rupture at 12 months follow-up

	Group A (bundle-bundle repair)	Group B (modified Bunnel suture)	Mean difference	95% confidence interval	t	*p*
Follow-up (months)	23.73 ± 2.81	22.61 ± 3.96	1.120	(− 0.874,3.114)	t = 1.124	*p* = 0.265
Rerupture rate	0	0	–	–	–	–
Dorsiflexion(°)	19.06 ± 2.42	19.25 ± 3.24	−0.195	(−1.663,1.273)	t = −2.66	*p* = 0.791
Plantflexion(°)	36.58 ± 4.39	35.41 ± 4.45	1.177	(−1.088,3.442)	t = 1.040	*p* = 0.303
Heel-rise (uninjured vs. injuried)	1.74 ± 0.96^*^	2.37 ± 1.42^*^	−1.178	(−1.170,-0.587)	t = −3.988	*p* = 0.000
Calf muscle circumference (uninjured vs. injuried)	1.74 ± 0.94^″*”^	2.34 ± 1.45^″*”^	−0.631	(−1.255,-0.007)	t = −2.025	*p* = 0.043
Anteroposterior diameter(mm)	10.16 ± 2.04	9.94 ± 2.01	0.215	(−0.825,1.255)	t = 0.413	*p* = 0.681
Cross-sectional diameter(mm)	16.54 ± 1.55^*^	18.49 ± 1.59^*^	−1.947	(−2.751,-1.143)	t = −4.847	*p* = 0.000
Length(cm)	11.98 ± 1.64	11.11 ± 1.74	0.874	(0.007,1.740)	t = 2.017	*p* = 0.048

^“*”^: statistically significant difference between two groups (*P* > 0.05; Student’s t-test)

**Table 3:** Results of the postoperative score evaluation in both groups at 12 months follow-up

	Group A(bundle-bundle repair)	Group B modified Bunnel suture)	Mean difference	95% confidence interval	t	*p*
ATRS	90.67 ± 2.66	91.10 ± 2.24	−0.496	(−1.688,0.828)	t = −0.065	*p* = 0.684
AOFAS	95.40 ± 3.65	95.38 ± 3.44	0.013	(−1.805,1.831)	t = −0.014	*p* = 0.989
VAS	0.36 ± 0.49	0.32 ± 0.48	0.044	(−0.203,0.291)	t = 0.357	*p* = 0.723

^“*”^: statistically significant difference between two groups (*P* > 0.05; Student’s t-test)

*ATRS* Achilles Tendon Total Rupture Score, *VISA-A* Victorian Institute of Sports Assessment-Achilles score, *VAS* Visual analogue scale score

## Discussion

The present study found that patients with acute Achilles tendon rupture achieved a better clinical outcome regarding the heel-rise test and calf circumference after surgical treatment using the bundle-to-bundle suture technique compared with the modified Bunnell suture technique. However, the two operative techniques achieved similar PROMs.

Rebeccato et al. [21] reported a 2% (− 0.67 cm) reduction in calf circumference on the injured side versus the uninjured side after surgery for Achilles tendon rupture. Furthermore, the postoperative calf volume of the affected leg was 91% of the volume of the healthy side [21]. In the present study, the calf circumference was smaller on the injured side than the uninjured side in both the percutaneous and open repair groups. In the final evaluation, the percutaneous repair group had a greater reduction in calf circumference than the open repair group. The ability to lift the heel by > 5 cm is considered to indicate normal Achilles tendon strength [18]. Haji et al. [17] reported that more patients who underwent percutaneous repair of Achilles tendon rupture were able to perform normal heel rises compared with those who underwent open surgery (92% vs. 83%, respectively). In the present study, the heel-rise test results were comparable at 12 months after the bundle-to-bundle suture technique and modified Bunnell suture technique (heel-rise index of 1.74 vs. 2.37, respectively). Clinical evaluation of the calf circumference at final follow-up demonstrated that the bundle-to-bundle suture technique produced a better result than the modified Bunnell suture technique. These results suggest that the bundle-to-bundle suture technique more effectively restores muscle strength than the modified Bunnell suture technique; this may be due to the absence of a gap at the tendon rupture site after bundle-to-bundle suturing, which enhances tendon healing.

In patients with Achilles tendon rupture, bundle-to-bundle suturing is the most effective surgical method to restore the anatomical structure and physiological characteristics of the tendon [4–6]. Open repair enables direct repair of the rupture site and achievement of maximum mechanical stability [4–7, 22]; however, the complex sutures used in the modified Bunnell suture technique can form a fiber block of the Achilles tendon that may result in keloid formation and tendon shortening [7, 12, 23–26]. Open repair also reportedly causes scar contracture of the Achilles tendon [23]. In contrast, repair of the ruptured Achilles tendon with the bundle-to-bundle suture technique prevents thickening and adhesion of the tendon [7]. Gigante et al. [6] found that the anteroposterior and cross-sectional diameters of the Achilles tendon did not significantly differ at 12 months after percutaneous repair compared with open repair. In the present study, the anteroposterior diameter of the Achilles tendon was significantly larger in the open repair group than in the percutaneous repair group. Therefore, the bundle-to-bundle suture technique more effectively reduced adhesion and hypertrophy of the Achilles tendon compared with the modified Bunnell suture technique.

Percutaneous repair of Achilles tendon rupture reportedly better retains the ankle range of motion than open repair, especially regarding the maximum dorsiflexion angle [5, 17]. However, the present study showed no difference in the ankle range of motion at the final evaluation after open versus percutaneous repair of Achilles tendon rupture. The Achilles tendon was longer in the bundle-to-bundle suture group than in the modified Bunnell suture group, but this had no effect on the ankle range of motion and did not weaken the Achilles tendon strength. The reason for this may be that the anatomical length of the Achilles tendon was restored, and the fibers were more tightly connected after repair using the bundle-to-bundle suture technique.

The present study also found no difference between the two groups in the clinical function scores (ATRS, AOFAS score, and VAS score) at 12 months postoperatively. In previous studies, the AOFAS score ranged from 96.3 to 96.8 after percutaneous repair, and from 96.1 to 98.7 after open procedures [24, 25]. In the present study, the postoperative AOFAS score significantly improved in both groups to 95.40 ± 3.65 in the open repair group, and 95.38 ± 3.44 in the percutaneous repair group. However, there was no significant difference between groups in the mean VAS score at 12 months postoperatively (1.6 in the percutaneous repair group vs. 1.7 in the open repair group).

Open repair can result in complications such as deep and superficial infection and poor wound healing [25, 26]. The aim of percutaneous repair is to minimize the risk of complications [26–28]. Although percutaneous repair has previously been associated with a high rate of nerve injury (2.9%) [5], there was no nerve injury in either group in the present study. The most frequent complication of open repair is poor surgical wound healing, because of the creation of a longitudinal incision in poorly vascularized skin [29]. In the present study, the incidence of poor wound healing was higher in the open repair group than in the percutaneous repair group. However, there was only one case of delayed wound healing, and no bacterial infection developed. These findings are consistent with a systematic review that showed that the rate of wound infection is significantly lower after percutaneous puncture than open surgery [11]. In the bundle-to-bundle suture technique of the present study, we used a medial incision, removed the blood clots, and preserved the aponeurosis to reduce the risk of poor postoperative wound healing.

Several limitations must be considered when interpreting the results of the present study. First, there were fewer females than males, so it remains unclear whether the outcomes of the two techniques differ between sexes. Second, all patients in the present study were < 45 years old; thus, it remains unclear whether the bundle-to-bundle suture technique is applicable to patients aged ≥45 years. Randomized controlled trials may be required to identify the optimal surgical method for acute Achilles tendon rupture. Third, the strength of the Achilles tendon was assessed using the heel-rise index (an infrequently described outcome evaluation), rather than via direct measurement with a specific instrument.

Fourth, the present sample size was small, and outliers might have had a significant influence on the results. No sample size calculation was made, and the study protocol was not registered prior to study initiation. Fifth, the calculated *p* values were not corrected for multiple testing, which increases the risk of false positive results. Sixth, the follow-up duration was relatively short; further follow-up is needed to evaluate the long-term effects.

## Conclusions

The present study found that patients with acute Achilles tendon rupture treated with bundle-to-bundle repair achieved a better clinical outcome regarding the heel-rise test that is an infrequently described outcome evaluation and calf circumference compared with patients treated with the modified Bunnell suture technique. However, the PROMs did not differ between the two treatment groups.

## Data Availability

The datasets generated and/or analyzed during the current study are not publicly available because they contain patients’ personal information. However, these datasets are available from the corresponding author on reasonable request.

## Abbreviations

ATRS: Achilles tendon total rupture score
AOFAS: American Orthopaedic Foot and Ankle Society
VAS: Visual analog scale
MRI: Magnetic resonance imaging
BMI: Body mass index

## References

[1] Maganaris CN, Narici MV, Maffulli N (2008). Biomechanics of the Achilles tendon. Disabil Rehabil.

[2] Longo U, Petrillo S, Maffulli N, Denaro V (2013). Acute achilles tendon rupture in athletes. Foot Ankle Clin.

[3] Houshian S, Tscherning T, Riegels-Nielsen P (1998). The epidemiology of Achilles tendon rupture in a Danish county. Injury.

[4] Liechti DJ, Moatshe G, Backus JD, Marchetti DC, Clanton TO (2018). A percutaneous knotless technique for acute Achilles tendon ruptures. Arthrosc Tech.

[5] Del Buono A, Volpin A, Maffulli N (2014). Minimally invasive versus open surgery for acute Achilles tendon rupture: a systematic review. Br Med Bull.

[6] Gigante A, Moschini A, Verdenelli A, Del Torto M, Ulisse S, de Palma L (2008). Open versus percutaneous repair in the treatment of acute Achilles tendon rupture: a randomized prospective study. Knee Surg Sports Traumatol Arthrosc.

[7] Li CG, Li B, Yang YF (2017). Management of acute Achilles tendon rupture with tendon-bundle technique. J Int Med Res.

[8] Guillo S, Del Buono A, Dias M, Denaro V, Maffulli N (2013). Percutaneous repair of acute ruptures of the tendo Achillis. Surgeon.

[9] Khan M, Li Z, Wang J (2005). Repeated exposure of tendon to prostaglandin-E2 leads to localized tendon degeneration. Clin J Sport Med.

[10] Rowley DI, Scotland TR (1982). Rupture of the Achilles tendon treated by a simple operative procedure. Injury.

[11] Li Q, Wang C, Huo Y, Jia Z, Wang X (2016). Minimally invasive versus open surgery for acute Achilles tendon rupture: a systematic review of overlapping meta-analyses. J Orthop Surg Res.

[12] Nyyssonen T, Saarikoski H, Kaukonen JP, Luthje P, Hakovirta H (2003). Simple end-to-end suture versus augmented repair in acute Achilles tendon ruptures: a retrospective comparison in 98 patients. Acta Orthop Scand.

[13] Barber FA, McGarry JE, Herbert MA, Anderson RB (2008). A biomechanical study of Achilles tendon repair augmentation using GraftJacket matrix. Foot Ankle Int.

[14] Zhao J, Yu B, Xie M, Huang R, Xiao K (2016). An alternative bundle-to-bundle suturing technique for repairing fresh Achilles tendon rupture. J Foot Ankle Surg.

[15] THOMPSON T, DOHERTY J (1962). Spontaneous rupture of tendon of Achilles: a new clinical diagnostic test. J Trauma.

[16] Ma GW, Griffith TG (1977). Percutaneous repair of acute closed ruptured achilles tendon: a new technique. Clin Orthop Relat Res.

[17] Haji A, Sahai A, Symes A, Vyas J (2004). Percutaneous versus open tendo achillis repair. Foot Ankle Int.

[18] Becher C, Donner S, Brucker J, Daniilidis K, Thermann H (2018). Outcome after operative treatment for chronic versus acute Achilles tendon rupture - A comparative analysis. Foot Ankle Surg.

[19] Nilsson-Helander K, Thomeé R, Silbernagel KG, Thomeé P, Faxén E, Eriksson BI, Karlsson J (2007). The Achilles tendon Total rupture score (ATRS): development and validation. Am J Sports Med.

[20] Kitaoka H, Alexander I, Adelaar R, A Nunley J, Myerson M, Sanders M, Lutter L (1997). Clinical rating Systems for the Ankle-Hindfoot, midfoot, hallux, and lesser toes. Foot Ankle Int.

[21] Rebeccato A, Santini S, Salmaso G, Nogarin L (2001). Repair of the achilles tendon rupture: a functional comparison of three surgical techniques. J Foot Ankle Surg.

[22] Rozis M, Benetos IS, Karampinas P, Polyzois V, Vlamis J, Pneumaticos SG (2018). Outcome of percutaneous fixation of acute Achilles tendon ruptures. Foot Ankle Int.

[23] Moller M, Movin T, Granhed H, Lind K, Faxen E, Karlsson J (2001). Acute rupture of tendon Achillis. A prospective randomised study of comparison between surgical and non-surgical treatment. J Bone Joint Surg (Br).

[24] Cretnik A, Kosanovic M, Smrkolj V (2005). Percutaneous versus open repair of the ruptured Achilles tendon: a comparative study. Am J Sports Med.

[25] Aktas S, Kocaoglu B (2009). Open versus minimal invasive repair with Achillon device. Foot Ankle Int.

[26] Hsu AR, Jones CP, Cohen BE, Davis WH, Ellington JK, Anderson RB (2015). Clinical outcomes and complications of percutaneous Achilles repair system versus open technique for acute Achilles tendon ruptures. Foot Ankle Int.

[27] Haytmanek CT, Williams BT, Civitarese DM, Turnbull TL, Massey MB, Wijdicks CA, LaPrade RF, Clanton TO (2015). A biomechanical comparison of an open repair and 3 minimally invasive percutaneous Achilles tendon repair techniques during a simulated, progressive rehabilitation protocol. Am J Sports Med.

[28] McMahon SE, Hing CB, Smith TO (2011). A meta-analysis of randomised controlled trials comparing conventional to minimally invasive approaches for repair of an Achilles tendon rupture. Foot Ankle Surg.

[29] Haertsch P (1981). The blood supply to the skin of the leg: a post-mortem investigation. Br J Plast Surg.

